# The role of lncRNA H19 in tumorigenesis and drug resistance of human Cancers

**DOI:** 10.3389/fgene.2022.1005522

**Published:** 2022-09-27

**Authors:** Xun Zhang, Mingpeng Luo, Jiahang Zhang, Bize Guo, Shreya Singh, Xixi Lin, Hanchu Xiong, Siwei Ju, Linbo Wang, Yulu Zhou, Jichun Zhou

**Affiliations:** ^1^ Department of Surgical Oncology, The Sir Run Run Shaw Affiliated Hospital, Zhejiang University, Hangzhou, China; ^2^ Key Laboratory of Cancer Prevention and Intervention, Ministry of Education, Hangzhou, China; ^3^ Zhejiang University School of Medicine, Hangzhou, China; ^4^ The First Affiliated Hospital of Zhejiang Chinese Medical University, Hangzhou, China

**Keywords:** lncRNA H19, drug resistance, tumorigenesis, miRNA, chemotherapy, endocrine therapy, targeted therapy

## Abstract

Systemic therapy is one of the most significant cancer treatments. However, drug resistance often appears and has become the primary cause of cancer therapy failure. Regulation of drug target, drug metabolism and drug efflux, cell death escape (apoptosis, autophagy, et al.), epigenetic changes, and many other variables are complicatedly involved in the mechanisms of drug resistance. In various types of cancers, long non-coding RNA H19 (lncRNA H19) has been shown to play critical roles in tumor development, proliferation, metastasis, and multiple drug resistance as well. The efficacy of chemotherapy, endocrine therapy, and targeted therapy are all influenced by the expression of H19, especially in breast cancer, liver cancer, lung cancer and colorectal cancer. Here, we summarize the relationship between lncRNA H19 and tumorigenesis, and illustrate the drug resistance mechanisms caused by lncRNA H19 as well. This review may provide more therapeutic potential targets for future cancer treatments.

## 1 Background

Cancer is a global public health epidemic and is predicted to be the leading cause of death in 2018 according to the World Health Organization (WHO). As a result, research on cancer treatment has gained growing attention (World Health Organization, 2018). Systemic therapy is an important way of treating cancer, among many treatment interventions. However, drug resistance has become a major problem in current cancer recurrence and clinical treatment failure (Holohan et al., 2013), (Chen et al., 2017a). Two forms of drug resistance (intrinsic and acquired) can significantly influence the efficacy of systemic therapy. Intrinsic resistance means that the resistance-mediating factors pre-exist in the bulk of tumor cells before systemic therapy is received (Longley and Johnston, 2005). Acquired drug resistance can be caused by mutations and other adaptive responses, such as the increased expression of therapeutic targets and the activation of alternative compensatory signaling pathways during treatment (Longley and Johnston, 2005). As tumors are increasingly recognized to be highly heterogeneous, drug resistance can occur through therapy-induced selection of a small subpopulation of resistant cells in the original tumor, and tumor cells can acquire cross-resistance to a wide variety of drugs (Kartal-Yandim et al., 2016).

Long noncoding RNAs (LncRNAs) are defined as a class of non-coding RNAs which consist of more than 200 nucleotides (Huarte, 2015). They do not encode any proteins but can be transcribed by RNA polymerase II like mRNAs (Mirzaei et al., 2021). As research deepen, more evidence has revealed the various functions of lncRNAs at chromatin, transcriptional and post-transcriptional levels (Mirzaei et al., 2022). According to the locations where lncRNAs function, they can be divided into nuclear lncRNAs and cytoplasmic lncRNAs. The nuclear lncRNAs participate in chromatin remodeling and modification, chromosomal looping, transcriptional modulation, and RNA processing; while cytoplasmic lncRNAs usually interact with mature mRNA and/or protein (Wang et al., 2017a). Based on the mechanisms above, lncRNAs have been identified to participate in a series of cellular processes including cell growth, proliferation, apoptosis, invasion, metastasis, and the regulation of gene expression, etc., Therefore, disturbances or impairment in lncRNA expression leads to emergence of pathological events, especially cancer (Ashrafizaveh et al., 2021). In different types of cancer cells, more and more lncRNAs like lncRNA H19 (here after, referred as H19), have been verified to engage in tumor development and drug resistance of systemic therapy (Qu et al., 2015).

In this review, we focus on the relation between H19 and tumorigenesis. Then we identify the drug resistant roles played by H19 in various cancers, such as breast cancers, hepatocellular carcinoma, bladder cancers, lung cancers, etc., Meanwhile, the possible association between H19 and various types of drugs is summarized. Finally, we address the functions performed by H19 in different forms of cell death and the possible directions of further research relevant to H19.

## 2 The mechanism of H19 in tumorigenesis

H19 was the first discovered lncRNA; it was firstly reported in 1991 by Bartolomei et al. (1991) and was shown to lack a common open reading frame (ORF). The *H19* gene is a well-known imprinted oncofetal gene, which locates on human chromosome 11p15.5 and encodes for a processed 2.3 kb RNA (Pachnis et al., 1984). As an imprinting gene, *H19* is maternally expressed and shares a common enhancer region with IGF2 (Insulin-like growth factor 2) gene which expresses the paternal allele (DeChiara et al., 1991). Without relevant encoding protein expression, H19 can be highly expressed in extraembryonic tissues, the embryo proper and most fetal tissues, but not expressed in most tissues postnatally (Matouk et al., 2007a). H19 has been described to be located in both cytoplasm and nucleus, although it was reported mainly in cytoplasm before (Schoenfelder et al., 2007), (Seidl et al., 2006). Recent evidence shows that the expression of *H19* can be reactivated during regeneration and tumorigenesis in adult tissue, indicating that H19 is probably related to the development and progression of tumor (Gabory et al., 2010). Further study demonstrates that *H19* displays a cell-dependent and/or tumor type-dependent function. However, it is found that H19 also shows a tumor suppressor function in teratocarcinomas and pituitary tumors (Yoshimizu et al., 2008), (Wu et al., 2018).

Therefore, it remains unclear whether H19’s functional role is tumor suppressive or oncogenic. The function of H19 is largely dependent on the type of cancer, the stage of tumor formation, and the level of molecular signaling pathway (Matouk et al., 2007b). There are several cancers with abnormal expression of *H19*: breast cancers, pancreatic cancers (Ma et al., 2014), choriocarcinomas (Arima et al., 1997), hepatocellular carcinomas (Ye et al., 2019), ovarian cancers (Tanos et al., 1999), and so on (Ariel et al., 2000a). Furthermore, it is shown that the poor prognosis of patients is correlated with overexpressed H19, especially in higher grades and invasive transitional cell carcinomas (Ariel et al., 2000b), (Ariel et al., 1995), (Gao et al., 2018).

**FIGURE 1:**
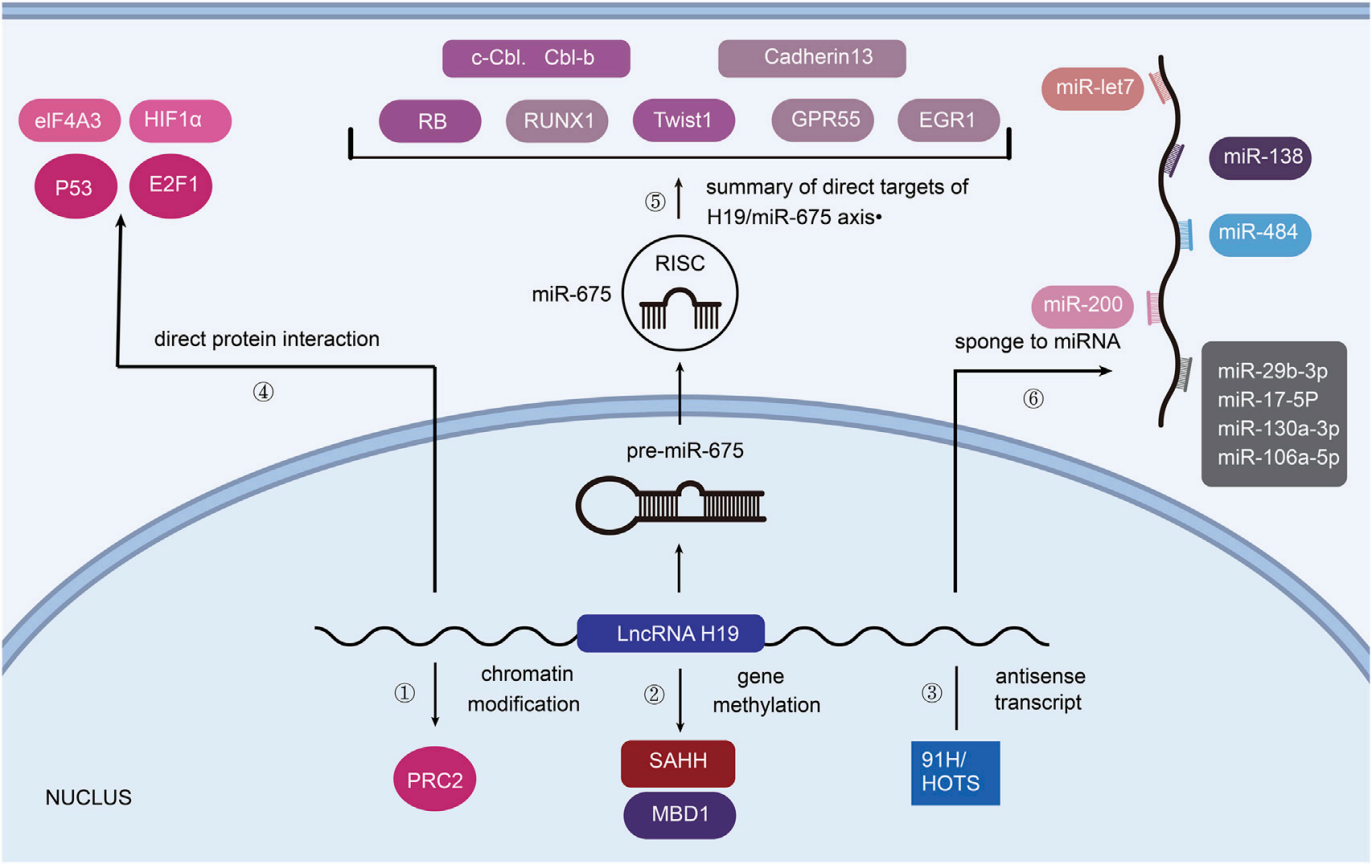
H19 associated mechanisms towards tumorigenesis. **①** PRC2 consists of core components (EZH2/EZH1, EED, SUZ12). **②** H19 binds SAHH or MBD1 to promote the methylation of imprinted genes. **③** 91H and HOTS are the antisense transcripts of H19. **④** p53, E2F1 and eIF4A3, HIF1α can directly interact with H19 to affect tumorigenesis. **⑤** H19 is the precursor of miR-675. **⑥** H19 acts on post-transcriptional control as ceRNA.

The molecular mechanisms between H19 and tumorigenesis, as shown in Figure 1, largely depend on the partners that H19 interacts with.

### 2.1 Chromatin modification

As reviewed by Callum Livingstone, the expression of IGF2 is associated with the development of various cancers (Livingstone, 2013). H19 and IGF2 are demonstrated to compete each other for binding enhancer. Thus, H19 could regulate the progression of cancer by changing the expression of IGF2 (Schmidt et al., 1999). What’s more, in bladder cancer, H19 has been found to interact with polycomb repressive complex 2 (PRC2) by associating with enhancer of zeste homolog 2 (EZH2), which leads to the silencing of the E-cadherin gene (Luo et al., 2013). In this way, increasing expression of H19 could downregulate E-cadherin (repressor of cell invasion and metastasis) and Nkd1 (inhibitor of Wnt/β-catenin signaling), causing the progression of cancer cells (Zhang et al., 2017) (Figure 1①).

### 2.2 Gene methylation

Our previous study suggested that H19 could bind to SAHH (S-Adenosylhomocysteine Hydrolase) and inhibit it, so as to catalyze SAH hydrolysis (Zhou et al., 2015) (Figure 1②). SAH affects cellular DNA methylating, which means that H19 may alter the methylation of DNA and lead to distinct tumorigenesis. (Martinez-leal et al., 2008). Besides, MBD1 (Methyl-CpG–Binding Domain Protein 1), the partner protein of H19, can induce methylation at H3K9me3 (lysine 9 of histone H3) to differentially methylated regions (DMRs) of correlated imprinted genes like IGF2, SLC38A4 (tumor suppressor in hepatocellular carcinoma) and PEG1 (Monnier et al., 2013), (Li et al., 2021).

### 2.3 Antisense transcript

LncRNA 91H is a novel H19 antisense RNA which was first revealed by Berteaux et al. (2008) LncRNA 91H contributes to the expression of IGF2, showing its oncogenic role in breast cancer cells. HOTS (*H19* opposite tumor suppressor), an *H19* antisense transcript, is confirmed to inhibit tumor growth in rhabdomyosarcoma and choriocarcinoma (Onyango and Feinberg, 2011) (Figure 1③).

### 2.4 Direct protein interaction

As the protein encoded by tumor suppressor gene *TP53*, p53 is reported to repress the expression of *H19* by binding to *H19* promoter (Lottin et al., 1998). E2F1 is a transcription activator of E2F family which helps to carry out cell cycle. Berteaux et al. (2005) elucidated that E2F1 could also bind to *H19* promoter, resulting in G1/S transition and cell proliferation of breast cancers. Moreover, by recruiting and directly binding to eIF4A3 (an RNA-binding protein), H19 promotes the growth of colorectal cancer. Similarly, hypoxia-inducible factor 1-alpha (HIF1α) can physically interact with H19, inducing smooth muscle cell apoptosis and abdominal aortic aneurysm development (Li et al., 2018). However, the tumorigenesis pathway influenced by the interaction between H19 and different kinds of protein is still under exploration (Han et al., 2016) (Figure 1④).

### 2.5 H19/miR-675 axis

The regulation of miR-675 by H19 is illustrated to be responsible for limiting placental growth before birth and the progression of different Cancers (Keniry et al., 2012) (Figure 1⑤). As Matouk et al. (2015) have summarized, H19 derived miR-675 can induce epithelial mesenchymal transition (EMT) and promote tumorigenesis in many cancer types. In detail, H19 serves as the precursor of miR-675 and promotes it to directly target c-Cbl and Cbl-b mRNA so as to decrease their expression, leading to sustained activation of AKT and ERK pathways as well as enhanced cell proliferation and migration in breast cancers both *in vitro* and *in vivo* (Vennin et al., 2015). Other targets of miR-675 in tumors contain: Retinoblastoma protein (RB, a tumor suppressor) in colorectal cancer (Tsang et al., 2010), Twist 1 (a key mediator in epithelial-mesenchymal transition) in hepatocellular cancer (Hernandez et al., 2013), Runt Domain Transcription Factor1 (RUNX1, a tumor suppressor) in gastric cancer (Zhuang et al., 2014), Cadherin 13 (a member of cadherin subfamily) in glioma (Shi et al., 2014a), G protein-coupled receptor (GPR55) in non-small cell lung cancer (He et al., 2015), early growth response protein1 (EGR1) in human liver cancer (Li et al., 2015). MiR-675 is also found to modulate p53 level during bladder cancer cell growth and colorectal tumor metastasis, though p53 is not a direct target of miR-675 (Liu et al., 2016), (Cen et al., 2019).

### 2.6 Sponge to miRNA

H19 can also function as ceRNA (competing endogenous RNAs) by antagonizing miRNAs (Angrand et al., 2015) (Figure 1⑥). As a molecular sponge, H19 modulates the function of let-7 family miRNA to promote the development of cancers such as pancreatic ductal adenocarcinoma (Kallen et al., 2013). Moreover, there are many other miRNAs which can be sponged by H19: 1) miR-200 family to suppress metastasis of hepatocellular carcinoma (Zhang et al., 2013), 2) miR-200a and miR-138 to promote EMT in colon cancer (Zhang et al., 2013), 3) miR-200b/c to mediate EMT and MET in breast cancer (Zhou et al., 2017a), 4) miR484 and miR29b-3p to promote cell viability and EMT in lung cancer (Zhang et al., 2018), (Liu et al., 2019), 5) miR‐130a‐3p and miR‐17‐5p to develop cardiac cancer (Jia et al., 2019), 6) miR-106a-5p to promote the growth of melanoma by upregulating E2F3 (a member of the E2F transcription factor family) expression (Luan et al., 2018).

## 3 H19 plays different roles in drug resistance of human cancers

### 3.1 Common mechanism of drug resistance

Resistance to drug therapy has always been a great barrier to overcoming cancer. Each antitumor agent interacts with cancer cells in its own specific way, and each tumor has its own specific characteristics that determine its tumor progression. Numerous drug-resisting mechanisms has arisen as the result of the interactions between different tumors and drugs (Vasan et al., 2019). Generally, by acting on the surface or entering the cells, curative drugs can function within the tumor cells and alter the micro-environment at the same time. Some tumors are intrinsically resistant to specific drug damage. As reviewed, the tumor intrinsic factors affecting drug resistance are mainly derived from the genetic, transcriptional or functional characteristics of tumor cells themselves (Kalbasi and Ribas, 2020). For example, some cancers overexpress multi-drug resistance protein1 (MDR1) without previous exposure to chemotherapeutic agents, thus possessing intrinsic drug resistance (Thomas et al., 2003). As for acquired drug resistance, the mechanisms can be split into five components at the cellular level.

Firstly, regulating drug uptake and efflux is an important way to establish drug resistance (Gottesman, 2002). ATP-binding cassette (ABC) family, including P-glycoprotein (P-gp), multi-drug resistance-associated protein1 (MRP1) and breast Cancer resistance protein (BCRP/ABCG2), is an important membrane transporter family. It can not only transport nutrients and other molecules, but also mediate the release of drugs (Fletcher et al., 2016). Secondly, compartmentalization of clinical cytotoxic agents apart from their cellular/tissue targets in lysosomes, autophagosomes, and other intercellular vesicles, will promote drug resistance in cancer (Feng et al., 2014). Thirdly, changes in drug targets and enhanced inactivation of drugs by affecting cell metabolism also play important roles in drug resistance (Bar-Zeev et al., 2017). Moreover, because the ultimate targets of many chemotherapeutic drugs are nuclear DNA, the repair of these DNA becomes one of the most well-known mechanisms of drug resistance in cancer. Nucleotide excision repair system (NER) and homologous recombination repair mechanisms (RRM) are two major DNA repair systems, which can be impaired by gene mutation and epigenetic silence (Mansoori et al., 2017). Finally, blocking cell death pathways has been found to possibly result in drug resistance (Pistritto et al., 2016). Since apoptosis is the main pathway of cell death induced by most anticancer drugs, the anti-apoptotic signaling pathways are always overactive in drug-resistant cells (Wong, 2011).

### 3.2 The role of H19 in therapy resistance of human cancers

**FIGURE 2:**
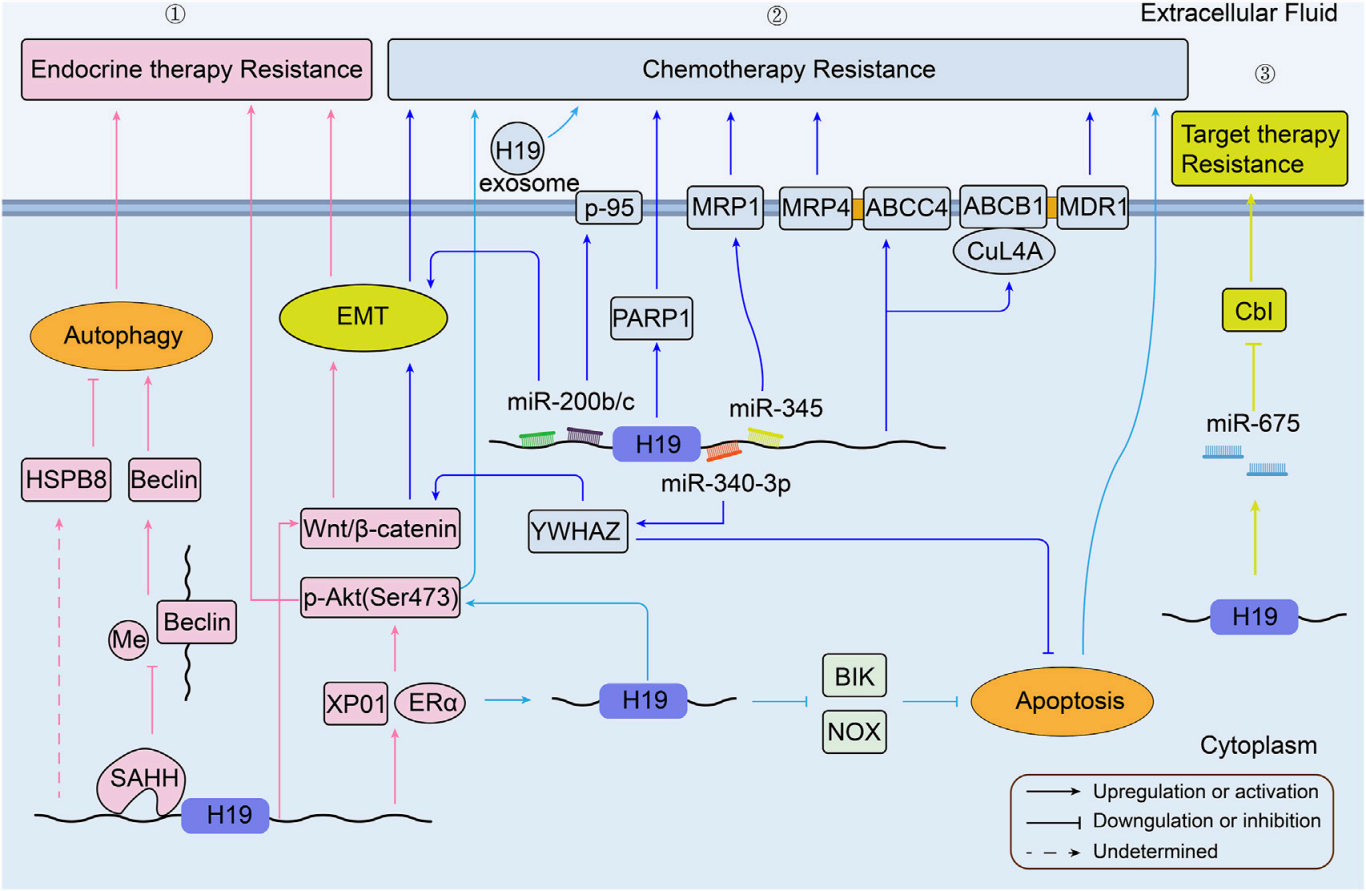
Overview of the role of H19 in modulating breast cancer therapy resistance. **①**H19 related pathways in BC endocrine therapy resistance; **②** H19 related pathways in BC chemotherapy resistance; **③** H19 related pathways in BC targeted therapy resistance (BC, breast cancer).

H19 has been shown to be involved in and expressed in almost every form of human cancers at all stages of tumorigenesis (Raveh et al., 2015). Chemotherapy, as well as endocrine therapy and targeted therapy, is one of the most effective approaches for the treatment of human cancers. Unfortunately, once drug resistance is established, these anti-cancer drugs cannot always kill tumor cells (Szakács et al., 2006). Although there are various molecular mechanisms for MDR, as shown in Figure 2, the pathways relevant to H19 still remain unclear in the occurrence of MDR. Researchers have so far confirmed several important roles that H19 plays in drug resistance of various cancers (Ghafouri-Fard et al., 2021), (Du et al., 2020). The role of H19 in the therapeutic resistance of human cancers are summarized in Table 1.

**TABLE 1:** Summary the drug resistance mechanisms to human cancers via H19.

Cancer type	Samples	Cell samples	Expression in resistant cell	Biological mechanism	Drugs	References
Breast cancer	—	MCF7, T47D, LCC2, LCC9	High	Increase of ERα protein expression	Tamoxifen, Fulvestrant	Basak et al. (2018)
	30 patients tissues	MCF7, SKBR3	High	Promotion of Wnt/β-catenin pathway and EMT process	Tamoxifen	Gao et al. (2018)
	BALB/c nude mouse/human	MCF7, MCF7/TAMR	High	Induction of autophagy activation via the *H19*/SAHH/DNMT3B axis		Wang et al. (2019)
	30 patients tissues		High	Mediate N-acetyltransferase 1 gene methylation		Sun et al. (2022)
	GEO database		High	Positive correlation with HSPB8		Gonzalez-Malerva et al. (2011)
		BT474, BT474/TAMR	High	Positive correlation with XPO1/ERα and promotion of Akt signaling		Kulkoyluoglu-Cotul et al. (2019)
	—	MCF7, MCF7/CDDP	No report	Sponge miR-200b/c to promote EMT, sponge miR-345 to upregulate MRP1	Cisplatin	Pogribny et al. (2010)
	—	MCF7, MCF7/DOXR	High	Increase of 95-kilodalton membrane glycoprotein (p95) expression	Doxorubicin	Doyle et al. (1996)
	BALB/c nude mouse/human, 63 pairs of BC and ANTs		High	H19-PARP1 pathway		Wang et al. (2020a)
	82 patients tissues		High	H19 delivery through exosomes		Wang et al. (2020b)
	—		High	Mediator of H19-CUL4A-ABCB1/ MDR1 and ABCC4/MRP4 pathway	Doxorubicin and paclitaxel	Zhu et al. (2017)
	—	MCF7, MCF7/PTXR, ZR751, ZR751/PTXR	High	Promotion of ERα-H19-BIK/NOXA signaling axis and apoptosis inhibition	Paclitaxel	Si et al. (2016)
	—	MCF7, MCF7/PTXR	High	H19/miR-340-3p/YWHAZ axis		Yan et al. (2020)
	BALB/c nude mouse	TNBC cell lines, MDA-MB-231/PTXR	High	Akt signaling pathway and deregulation of apoptotic regulatory proteins		Han et al. (2018)
	48 patients tissues	SKBR3, SKBR3/R	High	Down-regulation of Cbl through *H19*-derived miR-675	Trastuzumab	Sun et al. (2019a)
Hepatocellular carcinoma	—	HepG2, R-HepG2	High	Increase of MDR1/P-glycoprotein expression	Doxorubicin	Tsang and Kwok, (2007)
	42 patients tissues	CD133 + HuH7	High	Activation of MAPK/ERK signaling pathway and promotion of MDR1 and GST-π expression	Methotrexate	Ding et al. (2018)
	—	HepG2, HepG2/GEM	High	Up-regulation of CD90, CD44 and CD133 expression	Gemcitabine	ZJ, (2019)
	—	Bel-7402, HepG2, Hep3b, QGY- 7703, SMMC-7721	No report	Targeting PSEN1 through the H19/mir-193a-3p axis	Doxorubicin, paclitaxel, vinorelbine, 5-FU)	Ma et al. (2018)
				Promotion of PSEN1/γ-H2AX/Rad51	Radiotherapy (single-dose X-ray)	
	Mouse/Human, 32 patients tissues	HepG2, Plc/Prf5, and Huh7	Low	Increase of cytotoxic action and decrease of cell proliferation	Sorafenib, doxorubicin	Schultheiss et al. (2017)
	—	Huh7, HepG2	No report	Downregulation of miR-let-7 and overexpression of anti-apoptotic member Bcl-xL	Sorafenib	Shimizu et al. (2010)
	18 patients tissues	Huh7, Hep3B, SNU-449, SNU-387	High	H19/miR-675/EMT pathway	Sorafenib	Xu et al. (2020)
Lung cancer	—	HCC827, HCC827/R, HCC4006,HCC4006/R	High	Packaging H19 into exosomes	Gefitinib	Lei et al. (2018)
	BALB/c nude mouse	PC9, PC9/R, HCC827, HCC827/R	No report	Sponge miR-200c to activate Akt pathway and Bcl-2 and inhibit apoptosis		Zhou et al. (2017b)
				Overexpression of Akt and increased Cx26, promotion of EMT		Yang et al. (2015)
	BALB/c nude mouse	A549	No report	Downregulation of PTEN and PDCD4 and promotion of NFIB		Zhou and Zhang, (2020)
	—	PC9,PC9/R,HCC827,A529	High	Sponge miR-148b-3p to regulate DDAH1		Huang et al. (2019)
	Nude mouse/human, 65 patients tissues	PC9, PC9/ER, HCC827, HCC827/ER	Low	Interact with PKM2 and promote phosphorylation of AKT	Erlotinib	Chen et al. (2020)
	Nude mouse/human	HCC827, HCC827/ER, A549, A549/ER	High	Exosomal H19 and sponge miR-615-3p to up-regulate ATG7 expression and promote autophagy		Pan and Zhou, (2020)
	136 patients tissues	A549, A549/CDDP	High	H19 silencing induce apoptosis in cisplatin resistant cells	Cisplatin	Wang et al. (2017b)
	—	SK-MES-1	No report	High expression of GST-π		Wang et al. (2011)
Colorectal cancer	Nude mouse, 24 patients tissues	HT29, DLD1	High	Overexpression of H19-miR-675-5p axis and inhibition of VDR signaling	1,25(OH)2D3	Chen et al. (2017b)
	—	HT29, HT29/R	High	Activation of Wnt/β-catenin pathway	Methotrexate	feng Wu et al. (2017)
	Nude mouse	HCT116 and SW480	High	Exosomal H19 derived from CAFs, sponge of miR-141 and activation of β-catenin pathway	Oxaliplatin	Ren et al. (2018)
	110 patients tissues	HCT8, HCT8/R, HCT116 and SW1116	High	Induction of autophagy via *H19*/miR-194-5p/SIRT1, inhibition of apoptosis	5-FU	Wang et al. (2018a)
	-	LoVo	No report	H19-MDR1-MRP1-BCRP		Wang et al. (2018b)
	31 patients tissues	HCT116, DLD-1, SW480, HCT116/p, DLD-1/p, SW480/p	High	Down-regulation of RB and p27kip1		Yokoyama et al. (2019)
	30 patients tissues, Male athymic nude mice	HCT8, HCT116	No report	Sponge miR-200c to promote *JNK2* expression and ABCB1/P-gp	5-FU, pirabucin, cisplatin	Sui et al. (2014)
Gastric cancer	34 patients tissues	SGC7901, SGC7901/DDP	High	Target H19/miR-675 axis to suppress FADD mediated caspase8 and caspase3 dependent apoptosis	Cisplatin	Yan et al. (2017)
	39 patients tissues	MKN7	High	Promotion of H19/ IGF2BP3/PEG10 axis	Doxorubicin	Ishii et al. (2017)
	—	SGC7901, SGC7901/R	No report	Sponge miR-200bc/429 to modulate apoptosis	Vincristine	Zhu et al. (2012)
Neuronal glioma	69 patients tissues	U87, U87/R, U251,U251/R	High	Partly mediated by *MDR, MRP,* and *ABCG2*	Temozolomide	Jiang et al. (2016)
	61 patients tissues	U251,U251/R	No report	Regulation of MGMT expression		Xu et al. (2017)
Ovarian Cancer	Nude mouse, 54 patients tissues	A2780, A2780/DDP	High	Promotion of glutathione metabolism	Cisplatin	Zheng et al. (2016)
	—		High	Promote EZH2 expression and downregulate p21/PTEN		Sajadpoor et al. (2018)
	28 patients tissues		No report	Promotion of EMT transcription factors such as snail and slug		Haslehurst et al. (2012)
	—	OVCAR, OVCAR/DDP	High			Wu et al. (2019b)
Seminoma	BALB/c nude mouse, 20 patients tissues	TCam‐2, TCam-2/CDDP	High	Sponge miR‐106b‐5p and promote TDRG1 expression	Cisplatin	Wei et al. (2018)
Cardiac cancer	284 patients tissues	Human cardia cancer single‐cell suspension	High	Interact with miR‐130a‐3p and miR‐17‐5p	Cisplatin, doxorubicin, mitomycin, and 5-FU	Jia et al. (2019)
Choriocarcinoma	—	JEG‐3, JEG-3/MTXR, JEG-3/5-FUR	High	PI3K/ AKT/mTOR pathway	Methotrexate and 5‐FU	Yu et al. (2019)
Multiple myeloma	209 patients tissues	H929, U266, and 8226	High	Sponge miR-29b-3p to enhance MCL-1 and inhibit apoptosis	Bortezomib	Pan et al. (2019)
Laryngeal squamous cell carcinoma	60 patients tissues	TU-177, AMC-HN-8	High	H19/miR-107/HMGB1 axis and subsequent autophagy	Cisplatin	Chen et al. (2021)
Nasopharyngeal carcinoma	BALB/c nude mouse	NP69, C666-1, 6-10B	High	Inhibition of apoptosis	Doxorubicin, paclitaxel	Zhu, (2020)
Neuroendocrine prostate cancer	Biopsy tissues	LASCPC-01, NCI-H660 cell	High	Facilitate the PRC2 complex	Enzalutamide	Singh et al. (2021)

### 3.3 Breast cancer

Breast cancer is one of the most prominent and aggressive cancers in women (Israel et al., 2018). Female breast cancer has become the first commonly diagnosed cancer, with an estimated 2.3 million new cases (11.7%) in 2020 (Sung et al., 2021). H19 is involved in breast cancer cell growth, metastasis, and multiple drug resistance in different ways (Si et al., 2019), (Malhotra et al., 2017). A schematic illustration of the mechanisms by which H19 is involved in breast cancer therapy resistance is presented in Figure 2. In Tamoxifen-treated or Fulvestrant-treated estrogen receptor-alpha positive (ERα+) breast cancer tumors, high H19 expression is associated with increased drug resistance. H19 acts as an estrogen receptor modulator to promote the expression of ERα protein in endocrine therapy resistance (ETR) cells (Basak et al., 2018). Gao et al. (2018) found that knockdown of H19 could elevate tamoxifen sensitivity via Wnt/β-catenin pathway and EMT process in ER + breast cancers *in vitro*. Generally, tumors enhance autophagy activity to promote their metabolism and survival, to survive under microenvironmental stress, and to facilitate proliferation and aggressiveness (White, 2015). H19 activates autophagy via the downregulation of methylation in the promoter of Beclin1 by *H19*/SAHH/DNMT3B axis (SAHH and DNMT3B are two different sequences involved in tumor progression (Tan et al., 2017), (Sowińska et al., 2007). This process contributes to tamoxifen resistance (TAMR) in breast cancer (Wang et al., 2019). Moreover, N-acetyltransferase 1 (NAT1) was notably downregulated in MCF7/TAMR cell lines, but significantly elevated when knockdown H19. So it was possible that H19 conferred tamoxifen resistance via the mediation of NAT1 promoter methylation (Sun et al., 2022). Through analysis of gene functional groups, the expression of H19 is markedly higher in MCF7/TAMR cell lines (GSE26459). H19 has a positive correlation with heat shock protein family B (small) member 8 (HAPB8). And over-expression of HSPB8 may induce ETR through the regulation of autophagy (Gonzalez-Malerva et al., 2011). In another study with high H19 in BT474/TAMR (GSE112883), high exportin1 (XPO1) expression correlated with high ERα protein level, and high level of Akt signaling expression to help the tumor cell survive (Kulkoyluoglu-Cotul et al., 2019).

MiR-200 family was found to be sponged by H19 in several cancers, such as hepatocellular carcinoma (Zhang et al., 2013). Genomic analysis indicates that decreased miR-200b/c is associated with increased ZEB1 protein and the promotion of EMT in MCF7 cisplatin resistant cells (MCF7/CDDP). As reviewed above, H19 may sponge miR-345 to inhibit its expression, which upregulates MRP1 to promote cisplatin efflux in MCF7/CDDP (Pogribny et al., 2010). Apart from cisplatin, doxorubicin is another common drug tends to develop resistance to chemotherapy in breast cancer (Abe, 2005). It was reported that H19 induced 95-kilodalton membrane glycoprotein (p-95) expression to develop doxorubicin resistance in MCF-7 cells (Doyle et al., 1996). A new research revealed that H19 took part in the downregulated expression of Poly (ADP-ribose) polymerase (PARP)-1 to induce doxorubicin resistance both *in vitro* and in xenograft models (Wang et al., 2020a). Another study has also confirmed the H19 is over expressed in doxorubicin-resistant breast cancer cell subline compared with the matching parental cells. Additionally, H19 could be transferred from resistant cells to sensitive cells through exosomes, facilitating the chemoresistance of doxorubicin (Wang et al., 2020b). Upregulation of H19 has also enabled the chemoresistance of paclitaxel and anthracyclines analogues like doxorubicin in MCF-7 cells through H19-CUL4A-ABCB1/MDR1 (CUL4A, an ubiquitin ligase component; ABCB1, a member of the ATP-binding cassette family, which encodes MDR1) and ABCC4/MRP4 pathway (Zhu et al., 2017). In paclitaxel resistant cell line, the expression of ERα protein has a tight linkage with H19, suggesting that H19 is a downstream target of ERα. Associated with EZH2, H19 can downregulate the pro-apoptotic gene BIK and NOXA to inhibit apoptosis in ERα+ breast cancers (Si et al., 2016). Similarly, the over-expression of H19 has also been confirmed as an underlying therapeutic target in paclitaxel-resistant breast cancer cell subline. By binding with miR-340-3p, H19 subsequently regulates YWHAZ and potentiates the Wnt/β-catenin signaling. Such regulation can promote breast cancer cells’ proliferation, metastasis, and EMT features while inhibiting their apoptosis (Yan et al., 2020). Besides, H19 can also mediate Akt signaling pathway and inhibit apoptosis to make triple negative breast cancer (TNBC) resist to paclitaxel (Han et al., 2018). Another gene-expression group analysis shows high H19 in MCF7 methotrexate-resistant (MTXR) cell line and low H19 in MDA-MB-468/MTXR (GSE16080). In this study, over-expression of UGT1As was confirmed to induce methotrexate resistance in both breast cancer cell lines (Selga et al., 2009). More information about *H19* expression in epirubicin-resistant breast cancer cell lines is summarized in Supplementary Table S1.

Human epidermal growth factor receptor 2 (HER2)-positive breast cancer is another common breast cancer subtype, which can be treated by targeted drugs such as trastuzumab (Cameron et al., 2017). It was hypothesized that trastuzumab resistant HER2-positive breast cancer cells might be formed by downregulating Cbl through *H19*-derived miR-675 (Sun et al., 2019a).

### 3.4 Hepatocellular carcinoma

According to the Global Cancer Statistics 2020, primary liver cancer is now the sixth most commonly diagnosed cancer and the third leading cause of cancer death worldwide. HCC accounts for 75%–85% of all cases of liver cancer (Sung et al., 2021). During the progression of hepatocellular carcinoma, the expression level of H19 transcripts is found imbalanced high (Iizuka et al., 2004). It was reported that knockdown of H19 suppressed MDR1 expression and its transcript P-glycoprotein via regulating *MDR1* promoter methylation. This regulation resulted in the increased doxorubicin accumulation level and sensitized doxorubicin toxicity in R-HepG2 cells (Tsang and Kwok, 2007). Similarly, another study showed that the downregulation of H19 might block MAPK/ERK signaling pathway by inhibiting drug resistance genes *MDR1* and (glutathione-s-transferase-π) *GST-π.* H19 was shown to facilitate cell apoptosis and reduce the response of CD133+ HuH7 cells to chemotherapeutic drugs like methotrexate (MTX) (Ding et al., 2018). Moreover, in gemcitabine-resistant HepG2 cell line, H19 showed a close association with high expression of CD44, CD90, and CD133. These three proteins are HCC stem cell markers and predict worse prognosis of HCC (ZJ, 2019). Additionally, Ma et al. (2018) demonstrated that restrained expression of H19 and over-expression of miR-193a-3p enhanced the survival rate of hepatoma cell line when they were tolerant to chemotherapeutic agents [Doxorubicin, paclitaxel, vinorelbine, 5-fluorouracil (5-Fu)]. By targeting H19/miR-193a-3p axis, high expression of presenilin 1(PSEN1) increased γ- H2AX and Rad51 expression, and inducted radio-resistance to single-dose X-ray in HCC cells.

**FIGURE 3:**
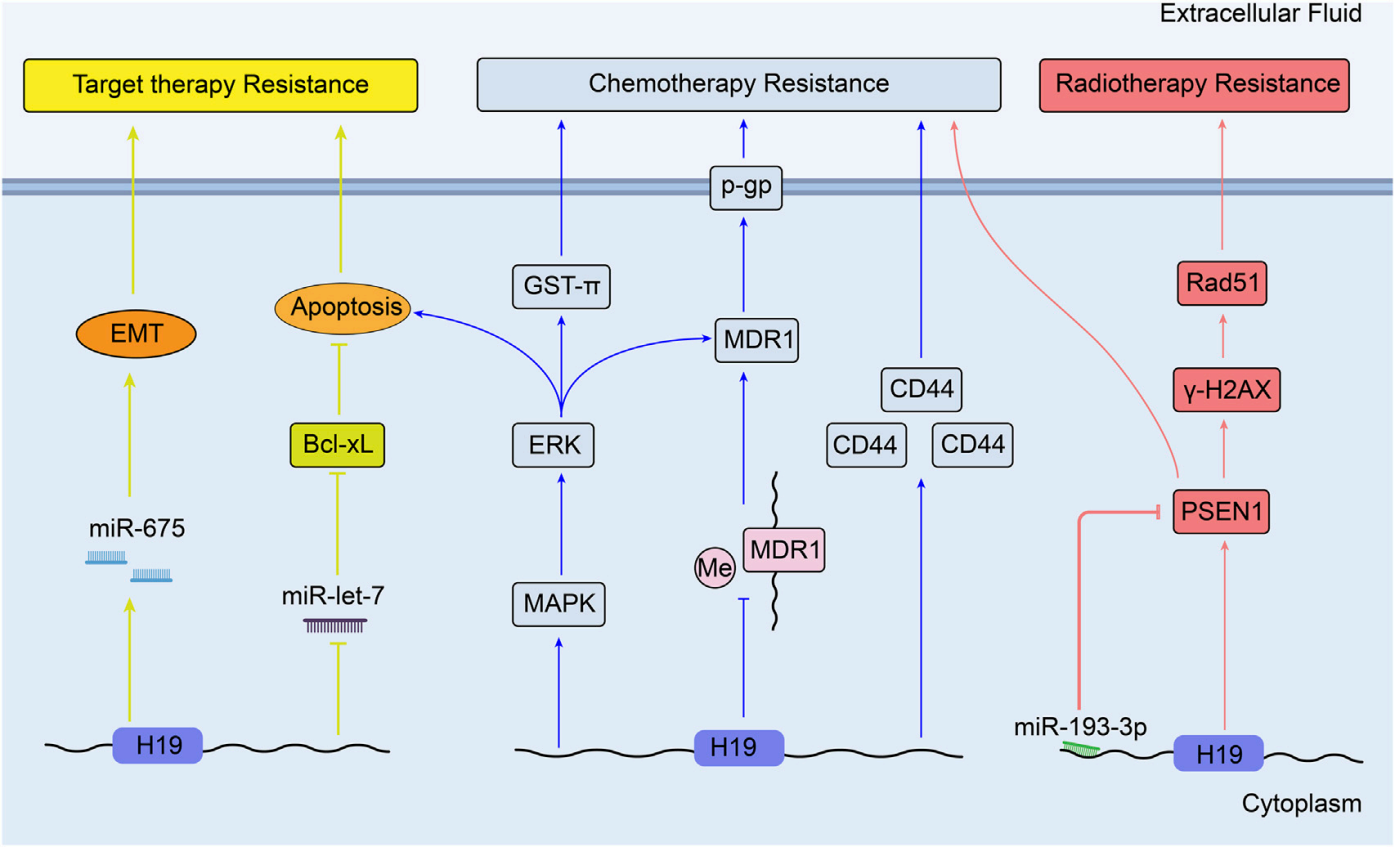
Overview of the role of H19 in modulating liver cancer therapy resistance.

Moreover, sorafenib was confirmed to induce apoptosis in HCC, which can be inhibited by potentiating anti-apoptotic member Bcl-xL expression. In human HCC tissues and cell line, low let-7 microRNA can enhance the expression of Bcl-xL and apoptosis (Shimizu et al., 2010). This finding suggests that the effect of sorafenib may be inhibited through high expression of H19 by sponging miR-let-7. Furthermore, it was newly found that upregulated H19/miR-675 expression could elevate sorafenib resistance by promoting EMT in HCC tissue samples and cells (Xu et al., 2020). However, the role of H19 in the therapy of HCC is not completely elucidated. In contrast, in chemo-resistant cells, over-expression of *H19* can reverse the drug resistance to doxorubicin, so that suppressing hepatocarcinogenesis and hepatoma cell growth (Schultheiss et al., 2017). Therefore, H19 has a dual effect on therapy resistance in HCC. Figure 3 shows the mechanisms of H19 in the therapy resistance of liver cancer.

### 3.5 Lung cancer

Lung cancer is the second most commonly diagnosed cancer and remains the leading cause of cancer death in 2020 (Sung et al., 2021). Currently, the impact of H19 on resistance to therapeutic option is mainly focused on non-small cell lung cancer (NSCLC). Such patients can benefit from the inhibitors of the epidermal growth factor receptor tyrosine kinase (EGFR TKIs), like gefitinib and erlotinib. (Kris et al., 2003). Lei et al. (2018) have proved that gefitinib resistance in NSCLC cells can be induced by packaging the H19 into exosomes and transferring it to these non-resistant cells. Zhou et al. (2017b) testified that miR-200c could enhance sensitivity of drug-resistant NSCLC to gefitinib by decreasing phosphorylated-Akt signaling and Bcl-2 expression. So it can be speculated that high expression of H19 induce gefitinib resistance through sponging miR-200c and inhibiting apoptosis. Similarly, it is proposed that the cooperation between PI3K/Akt pathway and connexin 26 (Cx26) can induce EMT and confer the gefitinib resistance of NSCLC cells (Yang et al., 2015). Thus, H19 silencing has been confirmed to increase the anticancer impacts of gefitinib in NSCLC through upregulation of PTEN and PDCD4 (both are tumor suppressors) and inhibition of nuclear factor I/B (NFIB) (Zhou and Zhang, 2020). Besides, H19 could also confer resistance to gefitinib via miR-148b-3p/dimethylarginine dimethylaminohydrolase-1 (DDAH1) axis in lung adenocarcinoma (Huang et al., 2019). Furthermore, unlike the usual correlation between high H19 expression and drug resistance, it is demonstrated that knockdown of H19 results in the resistance to erlotinib *in vivo* and *in vitro* by upregulating pyruvate kinase isoform muscle 2 (PKM2) expression and enhancing the phosphorylation of AKT (Chen et al., 2020). In contrast, upregulated H19 in erlotinib-resistant cells can sponge miR-615-3p to promote autophagy. Packaged exosomal H19 can also facilitate erlotinib resistance through miR-615-3p/ATG7 axis in NSCLC sensitive cells (Pan and Zhou, 2020).

**FIGURE 4:**
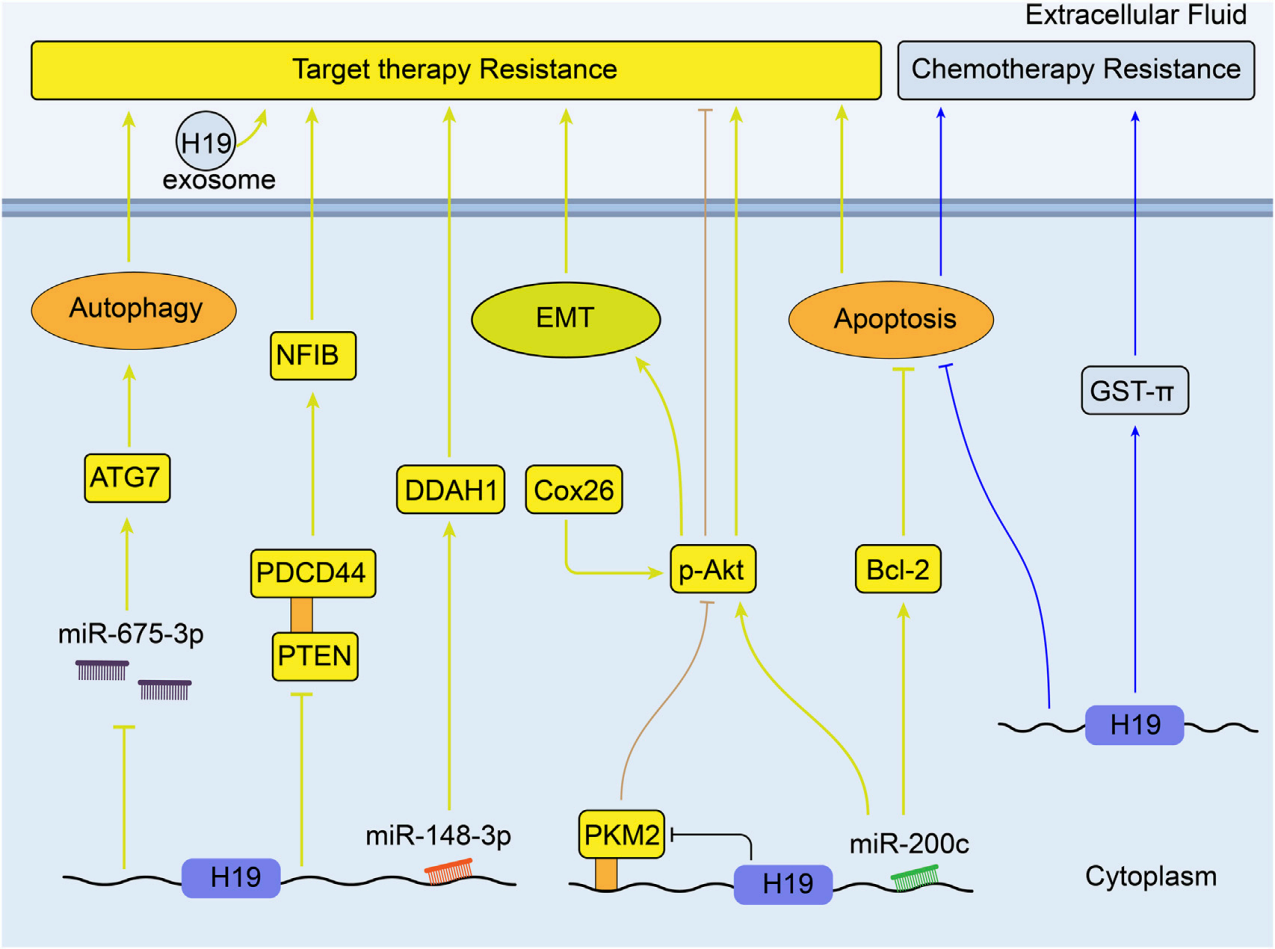
Overview of the role of H19 in modulating lung cancer therapy resistance.

H19 mediates the regulation of cisplatin resistance in human lung adenocarcinoma cells through apoptosis inhibition. Consistent with the results *in vitro*, over-expression of H19 is associated with worse clinical outcomes of patients who receive cisplatin-based therapy (Wang et al., 2017b). From other point, it has been discussed that H19 may promote gene *GST-π* expression in hepatocellular carcinoma (Ding et al., 2018). In another research, GST-π expression is reported to be positively correlated with the resistance to cisplatin in lung cancer cell lines, which means H19 may affect the lung cancer drug resistance through H19/ GST-π pathway (Wang et al., 2011). Figure 4 shows the mechanisms of H19 in the target therapy and chemotherapy resistance of lung cancer.

### 3.6 Colorectal cancer

**FIGURE 5:**
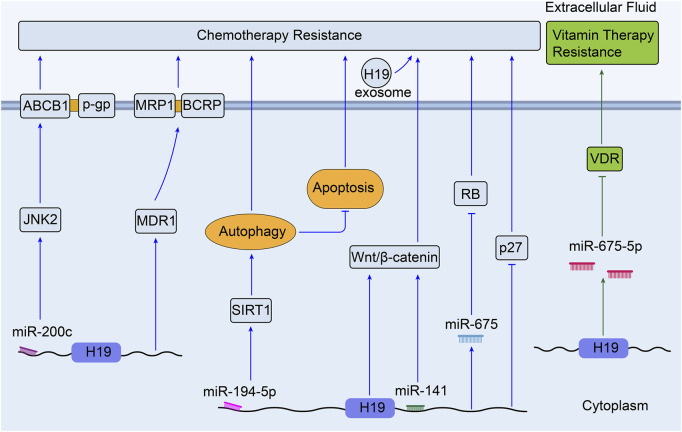
Overview of the role of H19 in modulating colorectal cancer therapy resistance.

Colorectal cancer is the third most commonly diagnosed cancer and the second most common cause of cancer-associated mortality over 185 countries (Sung et al., 2021). As reviewed, 1,25(OH)2D3 (the most active form of vitamin D in the human body) and its analogs have positive anti-tumor effect in colorectal cancer (Dou et al., 2016).S. Chen et al. have found that colon cancer cells show different resistance to the treatment of 1,25 (OH) 2D3 both *in vitro* and *in vivo* when H19 is overexpressed. They also discovered that H19 is able to downregulate the expression of Vitamin D receptor (VDR) by transcribing miR-675-5p, indicating the important role of *H19* underlying the development of resistance to 1,25 (OH) 2D3 treatment in advanced colon cancer cells (Chen et al., 2017b). Besides, chemotherapeutic resistance is a mainly formidable challenge in the treatment of colorectal cancer (Figure 5).

Methotrexate (MTX) is one of anti-metabolite and anti-folate chemotherapeutic agents for various cancers including CRC, and it is revealed that H19 can mediate MTX resistance by activating Wnt/β-catenin signaling in colorectal cancer cell line HT-29 (feng Wu et al., 2017). Through integrative bioinformatics analysis, H19 is observed to play key roles in the process of oxaliplatin or irinotecan resistance in colorectal cancer (Sun et al., 2019b). Meanwhile, H19 shows lower expression in oxaliplatin- and irinotecan-resistant CRC cell lines compared with the parental cells (GSE42387, Supplementary Table S1) (Jensen et al., 2015). Moreover, the exosomes derived from carcinoma-associated fibroblasts (CAFs) have been found to transfer H19 to CRC cells and induce oxaliplatin resistance *in vitro* and *in vivo*. Upregulated H19 can activate the Wnt/β-catenin pathway and promote the stemness of CRC cells through sponging miR-141 (Ren et al., 2018). What’s more, it was concluded that many lncRNAs including H19 could act as regulators of autophagy and participate in CRC drug resistance (Bermúdez et al., 2019). Wang et al. (2018a) confirmed that H19 could sponge miR-194-5p to promote autophagy via NAD-dependent deacetylase sirtuin-1(SIRT1), so that to enhance 5-Fu chemoresistance in CRC cells. H19 silencing decreased the expression of MDR1, MRP1, and BCRP, which could reverse the sensitivity to 5-Fu in CRC (Wang et al., 2018b). In 5-Fu resistant rectal cancer cells, H19 was linked with downregulation of RB and p27kip1 (p27, a tumor suppressor) (Yokoyama et al., 2019). In addition, miR-200c was found to reduce the expression of JNK2(a set of enzymes in response to a plethora of stress signals) gene and ABCB1 mediated P-gp; this sensitized the MDR colorectal cancer cells to chemotherapeutic drugs, like cisplatin, 5-FU, pirabucin (Sui et al., 2014). According to previous studies, H19 can potentially sponge miR-200c to regulate the process of MDR in CRC.

### 3.7 Gastric cancer

The incidence and mortality of gastric cancer (GC) have been both increasing dramatically in most countries worldwide during recent 30 years (Etemadi et al., 2020). Over-expression of H19 has been confirmed to be associated with anti-apoptotic and metastatic properties in gastric cancer, leading to multi-drug resistance of tumor (Li et al., 2014). Cisplatin-resistant gastric cancer cell line SGC7901 showed high expressions of H19/miR-675 and low expression of Fas-associated death domain (FADD), which suppressed caspase8 and caspase3 dependent apoptosis (Yan et al., 2017). What’s more, down-regulation of H19 was shown to reduce doxorubicin 50% inhibition concentration (IC50) and alleviate chemoresistance in GC cells. In this study, H19 can promote the expression of IGF2BP3(IGF2 mRNA binding protein 3) and PEG10 (Paternally Expressed 10) (Ishii et al., 2017). The miR-200 family can be divided into miR-200bc/429 cluster and miR-200a/141 cluster, and these two clusters function specifically on different cell types. In GC cell lines, miR-200bc/429 cluster could target X-linked inhibitor of apoptosis protein (XIAP) and BCL2 to modulate apoptosis, promoting the formation of vincristine (VCR) resistance (Dehghanzadeh et al., 2015), (Zhu et al., 2012).

### 3.8 Neuronal glioma

H19/miR-675 signaling plays a critical role in glioma progression (Shi et al., 2014b). It is the major determinant conferring oncogenic properties to the glioma cells. As reported, the over-expression of H19 can promote temozolomide (TMZ) resistance in glioma cell lines. Compared to the TMZ-sensitive tumors, the major drug resistance genes such as *MDR, MRP,* and *ABCG2* and their expressed mRNA and protein are found to upregulate in the TMZ-resistant (TMZR) glioma cell lines (Jiang et al., 2016). Through gene expression analysis between glioblastoma LN229 cell line and LN229/TMZR, H19 shows a lower expression in resistant cell line (GSE113510). The researchers focused on increased MGMT (O^6^-methylguanine-DNA methyltransferase) expression regulated by lncRNA TALC (temozolomide-associated lncRNA in glioblastoma recurrence) in TMZR cells (Wu et al., 2019a). Similar study in other temozolomide resistant glioma cells shows that H19 can confer temozolomide resistance by modulating MGMT expression (Xu et al., 2017). Additionally, via integrated bioinformatics analyses, Xiao et al. (2020) have found that H19’s copy number variations could affect the infiltration level of glioma immune cells. Consequently, H19 may be future target to the immunotherapy for glioma.

### 3.9 Ovarian cancer

Recently, it was shown that the expression of H19 was enhanced in cisplatin-resistant ovarian cancer cells. H19 can confer cisplatin resistance to ovarian cancer cells via regulating glutathione metabolism *in vitro* and *in vivo* (Zheng et al., 2016). Sajadpoor et al. (2018) confirmed that valproic acid (VPA) could negatively regulate the H19 and EZH2 expression in ovarian cancer A2780 cisplatin-resistant cells, which subsequently lead to cell apoptosis. Therefore, H19 could increase cisplatin resistance in ovarian cells by targeting EZH2/p21/PTEN pathway. Another research revealed that EMT transcription factors snail and slug contributed to cisplatin resistance in ovarian cancer, indicating the potential new mechanism between H19 and cisplatin resistance (Haslehurst et al., 2012). Downregulation of H19 can inhibit EMT, migration and sensibility of cisplatin in these cells (Wu et al., 2019b). Another studies about gene expression difference in ovarian cancer also showed high H19 expression in cisplatin and oxaliplatin resistant ovarian cancer cells (GSE28648) (Zeller et al., 2012). The role of miR-483-3p and modulated protein kinase C α(PKCα) was focused on the occurrence of drug resistance (GSE58472) (Arrighetti et al., 2016).

### 3.10 Other cancers

Moreover, H19 promotes the cisplatin resistance in seminoma, resulting from the increasing expression of TDRG1 (testis developmental related gene 1) by sponging miRNA‐106b‐5p (Wei et al., 2018). H19 targeting miR‐130a‐3p and miR‐17‐5p could increase overall survival of cardiac cancer cells treated with cisplatin, doxorubicin, mitomycin, and 5‐fluorouracil (5-FU), leading to the establishment of chemoresistance for cardiac cancer (Jia et al., 2019).

H19 is also related to the drug resistance of choriocarcinoma (CC). The resistance of CC cells to MTX and 5-FU could be reduced after H19 is depressed. By knocking out gene *H19*, the proliferative, migratory, and invasive ability can be decreased and the apoptosis can be increased in MTX/5-FU treated CC cells (Yu et al., 2019). Besides, H19 over-expression would induce bortezomib resistance in multiple myeloma by targeting MCL-1 via miR-29b-3p (Pan et al., 2019).

Laryngeal squamous cell carcinoma (LSCC) is a highly aggressive malignancy, accounting for approximately 90% of all laryngeal cancer (Siegel et al., 2015). Notably, expression of H19 has been shown to be increased in LSCC tissues and drug-resistant cells. The resistance to cisplatin is mediated via H19/miR-107/HMGB1 axis and subsequent autophagy (Chen et al., 2021).

In nasopharyngeal carcinoma, knockdown of H19 in drug-resistant cells significantly increases their chemoresistance through apoptosis promotion. When combined with paclitaxel, silencing H19 could enhance tumor inhibition *in vivo* (Zhu, 2020).

Neuroendocrine prostate cancer (NEPC) is a highly lethal subtype of prostate cancer with high expression of H19. By binding to PRC2, H19 induces epigenetic changes and promotes the association of H19 with EZH2. Knockdown of H19 was testified to re-sensitize NEPC to enzalutamide (Singh et al., 2021).

## 4 Discussion

### 4.1 H19 and different kinds of anti-tumor drugs

The three primary types of anti-tumor systemic treatment are chemotherapy, endocrine therapy, and targeted therapy. According to the mechanism of action on cancer cells, the chemotherapy drugs we commonly use are divided into four categories: Antimetabolites (like MTX, 5-FU), DNA alkylators (like cisplatin, oxaliplatin, temozolomide), Tubulin/microtubule inhibitors (like paclitaxel, vincristine), and DNA topoisomerase inhibitors (like doxorubicin, mitomycin, pirabucin) (Bailly et al., 2020). Endocrine therapy drugs can be divided into three types: Hormone replacement drugs (like 1,25(OH)2D3), Hormone elimination drugs, and Anti-hormone drugs (like fulvestrant, tamoxifen). Small molecule-targeted therapy drugs (such as sorafenib, gefitinib, bortezomib) and monoclonal antibody (such as trastuzumab) are typical targeted therapy drugs. Many of the above-mentioned drugs may develop tolerance when used in certain cancers (Table 2).

**TABLE 2:** Summary of different resistant drugs to human cancers via H19.

Therapy type	Drugs		Involved cancer(s)	Potential mechanism associated with H19	References
Chemo-therapy	Antimetabolites	Methotrexate	Hepatocellular carcinoma; Colorectal cancer	H19/MDR1; Wnt/β-catenin, PI3K/AKT/mTOR MAPK/ERK; Sponge miRNAs (Autophagy)	Jia et al. (2019)
		5-FU	Colorectal cancer		Ding et al. (2018)
					feng Wu et al. (2017)
					Wang et al. (2018a)
					Yu et al. (2019)
	DNA alkylators	Cisplatin	Breast cancer; Ovarian cancer; Seminoma; Cardiac cancer; Laryngeal squamous cell carcinoma	*EZH2/p21/PTEN* pathway; *MDR, MRP,* and *ABCG2*; β-catenin pathway; Sponge miRNAs (Apoptosis); H19/miR-107/HMGB1 axis (Autophagy)	Pogribny et al. (2010)
		Oxaliplatin	Colorectal cancer		Sajadpoor et al. (2018)
		Temozolomide	Neuronal glioma		Wei et al. (2018)
					Ren et al. (2018)
					Jiang et al. (2016)
					Chen et al. (2021)
	Tubulin/microtubule inhibitors	Paclitaxel	Breast cancer;	*H19-CUL4A-ABCB1*/ MDR1; H19/miR-340-3p/YWHAZ axis; H19-BIK/AKT; Sponge miR-200/429 (Apoptosis)	Zhu et al. (2017)
		Vincristine	Gastric cancer		Yan et al. (2020)
					Si et al. (2016)
					Han et al. (2018)
					Zhu et al. (2012)
	DNA topoisomerase inhibitors	Doxorubicin	Breast cancer; hepatocellular carcinoma; Cardiac cancer	*H19-CUL4A-ABCB1*/ MDR1/P-gp; Interact with miR‐130a‐3p and miR‐17‐5p; Sponge miR-200c	Jia et al. (2019)
		Mitomycin	Cardiac cancer		Doyle et al. (1996)
		Pirabucin	Colorectal cancer		Zhu et al. (2017)
					Tsang and Kwok, (2007)
					Sui et al. (2014)
Endocrine therapy	Hormone Replacement	1,25(OH)2D3	Colorectal Cancer	H19-miR-675-5p axis	Chen et al. (2017b)
	Hormone Elimination	—			
	Anti-hormone	Fulvestrant	Breast cancer	*H19*/SAHH/DNMT3B axis (Autophagy); Wnt pathway/EMT; Bind to PRC2	(Basak et al., 2018)
		Tamoxifen			Gao et al. (2018)
		Enzalutamide	Neuroendocrine prostate cancer		Singh et al. (2021)
Targeted therapy	Small molecule targeted therapy drugs	Sorafenib	Hepatocellular carcinoma	Sponge miRNAs/PI3K/AKT/EMT (Apoptosis); Interaction with PKM2/AKT	Shimizu et al. (2010)
		Gefitinib	Lung cancer		Zhou et al. (2017b)
		Erlotinib			(Yang et al., 2015)
		Bortezomib	Multiple myeloma		Huang et al. (2019)
					Chen et al. (2020)
					Pan et al. (2019)
	Monoclonal antibody	Trastuzumab	Breast cancer	H19-miR675-Cbl pathway	Sun et al. (2019a)

In chemoresistance, sponging miR-200b/c, miR-345, miR-340-3p to regulate the expression of membrane protein are important mechanisms of H19. Apoptosis intervention usually confers chemoresistance, while autophagy regulation often confers endocrine therapy resistance. H19-mediated Wnt/β-catenin and EMT signaling pathways show important roles in chemoresistance, endocrine therapy resistance and targeted therapy resistance. Particularly, encoding miR-675 shows targeted resistance phenotype in breast cancer and liver cancer. As the summary of potential mechanisms associated with H19 and different anti-tumor drugs, three common ways to promote MDR through H19 are proposed: gene methylation and nuclear epigenetic changes, miRNA control in cytoplasm, and direct association with certain protein/transcription factors (TFs) (Wang et al., 2020c).

### 4.2 H19 and different kinds of cell death

The research of cell death has always been closely interrelated with drug resistance research. Until now, the most widely-used classification of programmed cell death is consisted of apoptosis, necrosis, autophagy-associated cell death and ferroptosis (Tang et al., 2019). In previous studies, apoptosis is demonstrated to be the most common form of cell death in the regulation of H19. Gene methylation, miRNA regulation and direct protein interaction all play irreplaceable roles in apoptosis inhibition. Because most of the clinical therapeutic drugs induce cell death through apoptosis, silencing H19 will become a non-negligible method to increase drug efficacy or/and inhibit MDR. As another form of cell death reported frequently, autophagy can also be regulated by gene methylation and miRNA sponge. However, the researches of necrosis and ferroptosis in systemic therapy are scarce. In heart disease, necrosis is the main form of cardiomyocyte death. The miR-103/107-Fas-associated protein with death domain (FADD) pathway is demonstrated to induce necrosis in cardiac cell line H9c2. Consequently, H9c2 cells can be protected from necrosis by upregulating the expression of H19 (Wang et al., 2015). Although this finding was irrelevant to tumorigenesis and drug resistance, it revealed the possible relationship between H19 and cell necrosis.

As a new recognized regulated cell death first reported in 2012, ferroptosis gives rise to more and more researches, including those in anti-tumor therapy (Dixon et al., 2012). A recent study reported that inhibition of PI3K-AKT-mTOR signaling axis could sensitize breast cancer cells (BT474 and MDA-MB-453) to ferroptosis induction (Yi et al., 2020). Therefore, H19 may indirectly participate in ferroptosis by activating PI3K-AKT-mTOR signaling. Besides, the expression of iron storage protein ferritin is specifically dependent on H19/miR-675 expression levels.

Moreover, the interactive mechanism between ferritin and H19 differs in different cancer cells. It was found that the amounts of ferritin were negatively correlated with H19/miR675 levels in K562 cells (the first human established myelogenous leukemia cell line), but positively related in breast cancer cell line MCF7 cells (Di Sanzo et al., 2018). These researches develop a new level of interactive complexity between iron metabolism and H19 or some miRNAs expression. By regulating iron metabolism, ferroptosis will be broadly discussed in the cell death induction of H19.

### 4.3 Clinic and future prospect

Systemic therapy is one of the most important treatments for cancer patients. However, drug resistance has become the most urgent problem hampering our treatment. Along with the development of relevant studies, H19 has been testified to function in the tumorigenesis and drug resistance in human cancers via different mechanisms. Hence, increasing drug sensitivity and decreasing cancer cells drug resistance might be realized by targeting H19. Diphtheria toxin A-chain (DT-A)-H19 has shown anti-cancer effect by suppressing tumor growth in ovarian cancer (Mizrahi et al., 2009). DTA-H19 is a DNA plasmid that contains H19 gene regulatory sequences that drive the expression of an intracellular toxin. As an individualized DNA-based approach, DTA-H19 can be used in the tumors with high H19 expression. A phase 1/2a clinical trial for superficial bladder cancer has proved the therapeutic effect of intravesical DTA-19 (Sidi et al., 2008). Similar to H19, IGF2 is also highly active in various human cancers. The use of double promoter toxin vector H19-DTA-(IGF2)-P4-DTA exhibited superior inhibition towards pancreatic cancer, ovarian cancer, glioblastoma and HCC (Amit and Hochberg, 2012).

Nevertheless, more gene editing studies on H19 are still preclinical and are much needed. The regulation of cell death by H19 exerts a wide prospect of molecular research and clinical drug application. In the future, more attention needs to be paid to the additional functions and pathways related to H19, tumorigenesis and cells drug resistance. The research of H19 may provide us with a safer and more effective target to treat MDR and to enrich its function in genetics and molecular biology.
