# Supplementary material for: The role of lncRNA H19 in tumorigenesis and drug resistance of human Cancers
Source: Front Genet. 2022 Sep 27;13:1005522. doi: 10.3389/fgene.2022.1005522 (PMC9555214; doi:10.3389/fgene.2022.1005522)
Supplement: Supplementary file 1 [file Table1.DOCX]

| **Supplement Table 1** The H19 expression in different drug-resistant tumors | | | | | | | | | |
| --- | --- | --- | --- | --- | --- | --- | --- | --- | --- |
| Cancer type | Drug | Cell lines | Gene expression | Average | Expression in drug resistant cells | p value | Country | GEO Accession | Ref |
| Breast cancer | TAM | MCF7 B7TAMS-1 | 10.170 | 9.283 | High | p<0.05(*) | USA | GSE26459 | [1] |
|  |  | MCF7 B7TAMS-2 | 9.260 |  |  |  |  |  |  |
|  |  | MCF7 B7TAMS-3 | 8.420 |  |  |  |  |  |  |
|  |  | MCF7 G11TAMR-1 | 19.070 | 19.270 |  |  |  |  |  |
|  |  | MCF7 G11TAMR-2 | 15.830 |  |  |  |  |  |  |
|  |  | MCF7 G11TAMR-3 | 22.910 |  |  |  |  |  |  |
|  |  | BT474 TAMS-1 | 0.480 | 0.467 | High | p<0.05(*) | USA | GSE112883 | [2] |
|  |  | BT474 TAMS-2 | 0.450 |  |  |  |  |  |  |
|  |  | BT474 TAMS-3 | 0.470 |  |  |  |  |  |  |
|  |  | BT474 TAMR-1 | 1.130 | 1.037 |  |  |  |  |  |
|  |  | BT474 TAMR-2 | 1.130 |  |  |  |  |  |  |
|  |  | BT474 TAMR-3 | 0.850 |  |  |  |  |  |  |
|  | Methotrexate | MCF7 MTXS-1 | 640.300 | 678.933 | High | p<0.001(***) | Spain | GSE16070 | [3] |
|  |  | MCF7 MTXS-2 | 732.400 |  |  |  |  |  |  |
|  |  | MCF7 MTXS-3 | 664.100 |  |  |  |  |  |  |
|  |  | MCF7 MTXR-1 | 6347.700 | 5693.733 |  |  |  |  |  |
|  |  | MCF7 MTXR-2 | 5696.200 |  |  |  |  |  |  |
|  |  | MCF7 MTXR-3 | 5037.300 |  |  |  |  |  |  |
|  |  | MDA-MB-468 MTXS-1 | 116.850 | 119.883 | Low | p<0.001(***) | Spain | GSE16080 | [3] |
|  |  | MDA-MB-468 MTXS-2 | 138.350 |  |  |  |  |  |  |
|  |  | MDA-MB-468 MTXS-3 | 104.450 |  |  |  |  |  |  |
|  |  | MDA-MB-468 MTXR-1 | 26.250 | 16.217 |  |  |  |  |  |
|  |  | MDA-MB-468 MTXR-2 | 10.800 |  |  |  |  |  |  |
|  |  | MDA-MB-468 MTXR-3 | 11.600 |  |  |  |  |  |  |
|  | Epirubicin | MDA-MB-231-EPIS-1 | -0.780 | 1.109 | High | p<0.001(***) | Canada | GSE54326 | [4] |
|  |  | MDA-MB-231-EPIS-2 | -6.373 |  |  |  |  |  |  |
|  |  | MDA-MB-231-EPIS-3 | 10.480 |  |  |  |  |  |  |
|  |  | MDA-MB-231-EPIR-1 | 82.430 | 77.320 |  |  |  |  |  |
|  |  | MDA-MB-231-EPIR-2 | 65.040 |  |  |  |  |  |  |
|  |  | MDA-MB-231-EPIR-3 | 84.490 |  |  |  |  |  |  |
|  |  | MCF7 EPIS-1 | 1138.490 | 1146.337 | Low | p<0.05(*) |  |  |  |
|  |  | MCF7 EPIS-2 | 1168.080 |  |  |  |  |  |  |
|  |  | MCF7 EPIS-3 | 1132.440 |  |  |  |  |  |  |
|  |  | MCF7 EPIR-1 | 849.190 | 926.923 |  |  |  |  |  |
|  |  | MCF7 EPIR-2 | 989.260 |  |  |  |  |  |  |
|  |  | MCF7 EPIR-3 | 942.320 |  |  |  |  |  |  |
|  |  | SKBR3 EPIS-1 | 11272.280 | 11079.683 | High | p<0.05(*) |  |  |  |
|  |  | SKBR3 EPIS-2 | 11246.100 |  |  |  |  |  |  |
|  |  | SKBR3 EPIS-3 | 10720.670 |  |  |  |  |  |  |
|  |  | SKBR3 EPIR-1 | 15776.250 | 17192.327 |  |  |  |  |  |
|  |  | SKBR3 EPIR-2 | 18239.240 |  |  |  |  |  |  |
|  |  | SKBR3 EPIR-3 | 17561.490 |  |  |  |  |  |  |
|  |  | ZR75-1 EPIS-1 | -4.010 | -1.081 | NS | 0.161 |  |  |  |
|  |  | ZR75-1 EPIS-2 | -0.231 |  |  |  |  |  |  |
|  |  | ZR75-1 EPIS-3 | 0.997 |  |  |  |  |  |  |
|  |  | ZR75-1 EPIR-1 | -11.510 | -8.250 |  |  |  |  |  |
|  |  | ZR75-1 EPIR-2 | -11.860 |  |  |  |  |  |  |
|  |  | ZR75-1 EPIR-3 | -1.380 |  |  |  |  |  |  |
| Lung Cancer | gefitinib | HCC4006-GefS-1 | 84.090 | 83.193 | NS | 0.338 | Spain | GSE123066 | Missing |
|  |  | HCC4006-GefS-2 | 88.190 |  |  |  |  |  |  |
|  |  | HCC4006-GefS-3 | 77.300 |  |  |  |  |  |  |
|  |  | HCC4006-GefR-1 | 85.330 | 87.330 |  |  |  |  |  |
|  |  | HCC4006-GefR-2 | 91.080 |  |  |  |  |  |  |
|  |  | HCC4006-GefR-3 | 85.580 |  |  |  |  |  |  |
| Colon Cancer | MTX | HT29 MTXS-1 | 2.250 | 6.400 | NS | 0.703 | Spain | GSE11440 | [3], [5], [6] |
|  |  | HT29 MTXS-2 | 8.100 |  |  |  |  |  |  |
|  |  | HT29 MTXS-3 | 8.850 |  |  |  |  |  |  |
|  |  | HT29 MTXR-1 | 6.700 | 5.383 |  |  |  |  |  |
|  |  | HT29 MTXR-2 | 6.600 |  |  |  |  |  |  |
|  |  | HT29 MTXR-3 | 2.850 |  |  |  |  |  |  |
|  |  | Caco2 MTXS-1 | 10.600 | 9.450 | NS | 0.194 | Spain | GSE16066 | [3] |
|  |  | Caco2 MTXS-2 | 10.750 |  |  |  |  |  |  |
|  |  | Caco2 MTXS-3 | 7.000 |  |  |  |  |  |  |
|  |  | Caco2 MTXR-1 | 5.500 | 5.600 |  |  |  |  |  |
|  |  | Caco2 MTXR-2 | 2.150 |  |  |  |  |  |  |
|  |  | Caco2 MTXR-3 | 9.150 |  |  |  |  |  |  |
|  | oxaliplatin | HCT116 OXAS-1 | 8.290 | 8.227 | Low | p<0.01(**) | Denmark | GSE42387 | [7] |
|  |  | HCT116 OXAS-2 | 8.340 |  |  |  |  |  |  |
|  |  | HCT116 OXAS-3 | 8.050 |  |  |  |  |  |  |
|  |  | HCT116 OXAR-1 | 7.420 | 7.450 |  |  |  |  |  |
|  |  | HCT116 OXAR-2 | 7.650 |  |  |  |  |  |  |
|  |  | HCT116 OXAR-3 | 7.280 |  |  |  |  |  |  |
|  |  | HT29 OXAS-1 | 6.540 | 6.603 | Low | p<0.05(*) |  |  |  |
|  |  | HT29 OXAS-2 | 6.670 |  |  |  |  |  |  |
|  |  | HT29 OXAS-3 | 6.600 |  |  |  |  |  |  |
|  |  | HT29 OXAR-1 | 6.510 | 6.487 |  |  |  |  |  |
|  |  | HT29 OXAR-2 | 6.480 |  |  |  |  |  |  |
|  |  | HT29 OXAR-3 | 6.470 |  |  |  |  |  |  |
|  |  | LoVo OXAS-1 | 6.520 | 6.557 | High | p<0.05(*) |  |  |  |
|  |  | LoVo OXAS-2 | 6.630 |  |  |  |  |  |  |
|  |  | LoVo OXAS-3 | 6.520 |  |  |  |  |  |  |
|  |  | LoVo OXAR-1 | 7.540 | 7.557 |  |  |  |  |  |
|  |  | LoVo OXAR-2 | 7.850 |  |  |  |  |  |  |
|  |  | LoVo OXAR-3 | 7.280 |  |  |  |  |  |  |
|  | irinotecan | HCT116-IRIS-1 | 8.290 | 8.227 | Low | p<0.01(**) | Denmark | GSE42387 | [7] |
|  |  | HCT116-IRIS-2 | 8.340 |  |  |  |  |  |  |
|  |  | HCT116-IRIS-3 | 8.050 |  |  |  |  |  |  |
|  |  | HCT116-IRIR-1 | 7.540 | 7.533 |  |  |  |  |  |
|  |  | HCT116-IRIR-2 | 7.600 |  |  |  |  |  |  |
|  |  | HCT116-IRIR-3 | 7.460 |  |  |  |  |  |  |
| Ovarian Cancer | cisplatin | OVSAHO-CISS-1 | 8.170 | 8.330 | Low | p<0.05(*) | Italy | GSE93795 | [8] |
|  |  | OVSAHO-CISS-2 | 8.490 |  |  |  |  |  |  |
|  |  | OVSAHO-CISR-1 | 7.050 | 6.870 |  |  |  |  |  |
|  |  | OVSAHO-CISR-2 | 6.690 |  |  |  |  |  |  |
|  |  | A2780 CISS-1 | 5.320 | 5.500 | High | p<0.001(***) | UK | GSE28648 | [9] |
|  |  | A2780 CISS-2 | 5.420 |  |  |  |  |  |  |
|  |  | A2780 CISS-3 | 5.760 |  |  |  |  |  |  |
|  |  | CP70 CISR-1 | 9.140 | 9.510 |  |  |  |  |  |
|  |  | CP70 CISR-2 | 9.930 |  |  |  |  |  |  |
|  |  | CP70 CISR-3 | 9.460 |  |  |  |  |  |  |
|  |  | IGROV-1 CISS-1 | 244.350 | 293.800 | High | p<0.01(**) | Italy | GSE58472 | [10] |
|  |  | IGROV-1 CISS-2 | 362.050 |  |  |  |  |  |  |
|  |  | IGROV-1 CISS-3 | 275.000 |  |  |  |  |  |  |
|  |  | IGROV-1 CISR-1 | 730.480 | 716.767 |  |  |  |  |  |
|  |  | IGROV-1 CISR-2 | 711.820 |  |  |  |  |  |  |
|  |  | IGROV-1 CISR-3 | 708.000 |  |  |  |  |  |  |
|  | oxaliplatin | IGROV-1 OXAS-1 | 244.350 | 293.800 | High | p<0.05(*) |  |  |  |
|  |  | IGROV-1 OXAS-2 | 362.050 |  |  |  |  |  |  |
|  |  | IGROV-1 OXAS-3 | 275.000 |  |  |  |  |  |  |
|  |  | IGROV-1 OXAR-1 | 525.620 | 561.033 |  |  |  |  |  |
|  |  | IGROV-1 OXAR-2 | 690.750 |  |  |  |  |  |  |
|  |  | IGROV-1 OXAR-3 | 466.730 |  |  |  |  |  |  |
| Pancreatic Cancer | MTX | PaCa2 MTXS-1 | 45.750 | 60.000 | NS | 0.377 | Spain | GSE16082 | [3] |
|  |  | PaCa2 MTXS-2 | 64.950 |  |  |  |  |  |  |
|  |  | PaCa2 MTXS-3 | 69.300 |  |  |  |  |  |  |
|  |  | PaCa2 MTXR-1 | 49.300 | 77.267 |  |  |  |  |  |
|  |  | PaCa2 MTXR-2 | 82.150 |  |  |  |  |  |  |
|  |  | PaCa2 MTXR-3 | 100.350 |  |  |  |  |  |  |
| Erythtoblastic Leukemia | MTX | K562 MTXS-1 | 55.900 | 55.500 | Low | p<0.01(**) | Spain | GSE16085 | [3] |
|  |  | K562 MTXS-2 | 66.000 |  |  |  |  |  |  |
|  |  | K562 MTXS-3 | 44.600 |  |  |  |  |  |  |
|  |  | K562 MTXR-1 | 2.850 | 6.417 |  |  |  |  |  |
|  |  | K562 MTXR-2 | 10.800 |  |  |  |  |  |  |
|  |  | K562 MTXR-3 | 5.600 |  |  |  |  |  |  |
| Osteosarcoma | MTX | Saos-2 MTXS-1 | 10.150 | 5.383 | NS | 0.094 | Spain | GSE16089 | [3] |
|  |  | Saos-2 MTXS-2 | 4.500 |  |  |  |  |  |  |
|  |  | Saos-2 MTXS-3 | 1.500 |  |  |  |  |  |  |
|  |  | Saos-2 MTXR-1 | 30.150 | 21.017 |  |  |  |  |  |
|  |  | Saos-2 MTXR-2 | 10.600 |  |  |  |  |  |  |
|  |  | Saos-2 MTXR-3 | 22.300 |  |  |  |  |  |  |
| Gestational Trophoblastic Neoplasia | MTX | JEG-3 MTXS-1 | 6.290 | 8.243 | NS | 0.121 | USA | GSE88873 | Missing |
|  |  | JEG-3 MTXS-2 | 6.990 |  |  |  |  |  |  |
|  |  | JEG-3 MTXS-3 | 11.450 |  |  |  |  |  |  |
|  |  | JEG-3 MTXR-1 | 12.880 | 12.387 |  |  |  |  |  |
|  |  | JEG-3 MTXR-2 | 12.230 |  |  |  |  |  |  |
|  |  | JEG-3 MTXR-3 | 12.050 |  |  |  |  |  |  |
| Glioblastoma | temozolomide | LN229-TMZS-1 | 6.530 | 6.493 | Low | p<0.001(***) | China | GSE113510 | [11] |
|  |  | LN229-TMZS-2 | 6.470 |  |  |  |  |  |  |
|  |  | LN229-TMZS-3 | 6.480 |  |  |  |  |  |  |
|  |  | LN229-TMZR-1 | 4.480 | 4.457 |  |  |  |  |  |
|  |  | LN229-TMZR-2 | 4.390 |  |  |  |  |  |  |
|  |  | LN229-TMZR-3 | 4.500 |  |  |  |  |  |  |

**Reference**

[1] L. Gonzalez-Malerva *et al.*, “High-throughput ectopic expression screen for tamoxifen resistance identifies an atypical kinase that blocks autophagy,” *Proc Natl Acad Sci U S A*, vol. 108, no. 5, pp. 2058–2063, Feb. 2011, doi: 10.1073/PNAS.1018157108.

[2] E. Kulkoyluoglu-Cotul *et al.*, “Combined Targeting of Estrogen Receptor Alpha and XPO1 Prevent Akt Activation, Remodel Metabolic Pathways and Induce Autophagy to Overcome Tamoxifen Resistance,” *Cancers (Basel)*, vol. 11, no. 4, Apr. 2019, doi: 10.3390/CANCERS11040479.

[3] E. Selga, C. Oleaga, S. Ramírez, M. C. de Almagro, V. Noé, and C. J. Ciudad, “Networking of differentially expressed genes in human cancer cells resistant to methotrexate,” *Genome Med*, vol. 1, no. 9, Sep. 2009, doi: 10.1186/GM83.

[4] M. Braunstein *et al.*, “Downregulation of histone H2A and H2B pathways is associated with anthracycline sensitivity in breast cancer,” *Breast Cancer Res*, vol. 18, no. 1, Feb. 2016, doi: 10.1186/S13058-016-0676-6.

[5] N. Mencia, E. Selga, V. Noé, and C. J. Ciudad, “Underexpression of miR-224 in methotrexate resistant human colon cancer cells,” *Biochem Pharmacol*, vol. 82, no. 11, pp. 1572–1582, Dec. 2011, doi: 10.1016/J.BCP.2011.08.009.

[6] E. Selga, C. Morales, V. Noé, M. A. Peinado, and C. J. Ciudad, “Role of caveolin 1, E-cadherin, Enolase 2 and PKCalpha on resistance to methotrexate in human HT29 colon cancer cells,” *BMC Med Genomics*, vol. 1, no. 1, Dec. 2008, doi: 10.1186/1755-8794-1-35.

[7] N. F. Jensen *et al.*, “Establishment and characterization of models of chemotherapy resistance in colorectal cancer: Towards a predictive signature of chemoresistance,” *Mol Oncol*, vol. 9, no. 6, pp. 1169–1185, Jun. 2015, doi: 10.1016/J.MOLONC.2015.02.008.

[8] M. Sonego *et al.*, “Common biological phenotypes characterize the acquisition of platinum-resistance in epithelial ovarian cancer cells,” *Sci Rep*, vol. 7, no. 1, Dec. 2017, doi: 10.1038/S41598-017-07005-1.

[9] C. Zeller *et al.*, “Candidate DNA methylation drivers of acquired cisplatin resistance in ovarian cancer identified by methylome and expression profiling,” *Oncogene*, vol. 31, no. 42, pp. 4567–4576, Oct. 2012, doi: 10.1038/ONC.2011.611.

[10] N. Arrighetti *et al.*, “PKC-alpha modulation by miR-483-3p in platinum-resistant ovarian carcinoma cells,” *Toxicol Appl Pharmacol*, vol. 310, pp. 9–19, Nov. 2016, doi: 10.1016/J.TAAP.2016.08.005.

[11] P. Wu *et al.*, “Lnc-TALC promotes O6-methylguanine-DNA methyltransferase expression via regulating the c-Met pathway by competitively binding with miR-20b-3p,” *Nat Commun*, vol. 10, no. 1, Dec. 2019, doi: 10.1038/S41467-019-10025-2.
